# Detection and Quantification of Inorganic and Organic Anions in Natural, Potable, and Wastewaters in Northern New York Using Capillary Zone Electrophoresis and Indirect UV Detection

**DOI:** 10.4172/2157-7064.1000361

**Published:** 2017-05-30

**Authors:** Lara Varden, Britannia Smith, Fadi Bou-Abdallah

**Affiliations:** Department of Chemistry, State University of New York (SUNY) at Potsdam, 44 Pierrepont Avenue, Potsdam, NY, USA

**Keywords:** Capillary zone electrophoresis, Indirect UV detection, Organic and inorganic anions, Northern New York Raquette River, Adirondack watershed

## Abstract

Capillary zone electrophoresis (CZE) is a sensitive and rapid technique used for determining traces of inorganic and organic anions in potable, natural, and wastewaters. Here, CZE with indirect UV-diode array detection (CZE-DAD) was employed with a background electrolyte system comprising of an Agilent Technologies proprietary basic anion buffer at pH 12.0 and a forensic anion detection method. The limits of detection (LOD) for this method ranged between 3 and 5 ppm and involved hydrodynamic injection of 50 mbar for 6 s with a negative polarity separation voltage of −30 kV at 30°C, a detection wavelength of 350 nm and indirect reference of 275 nm. Fourteen different anions were checked for in the water samples that were examined and included bromide, chloride, thiosulfate, nitrate, nitrite, sulfate, azide, carbonate, fluoride, arsenate, phosphate, acetate, lactate, and silicate. The water samples were collected from Northern New York towns and the Raquette River water system, the third longest river in New York State and the largest watershed of the central and western Adirondacks. The concentrations detected for these anions ranged from <5.0 ppm to 260 ppm.

## Introduction

Clean water is vital for sustaining life. As demands on limited water resources continue to increase, new and efficient purification and detection methods are needed for water treatment, recovery and reuse. This is particularly important in the case of a natural disaster or chemical spills where untapped water sources including saltwater might become the only source of clean drinking water for millions of people. Our life is sustained by complex interactions of inorganic and organic substances. Nine essential inorganic ions play a substantial role in supporting and sustaining health and life including Na^+^, K^+^, Ca^2+^, Mg^2+^, H^+^, Cl^−^, HCO_3_^−^, PO_4_^3−^, and OH^−^, most of which are naturally found in our drinking water. Other non-essential ions with no known biological functions can be harmful to humans and wildlife if present above certain concentrations including lead (Pb), aluminum (Al), arsenic (As), barium (Ba), beryllium (Be), cadmium (Cd), gold (Au), lithium (Li), mercury (Hg), nickel (Ni), silver (Ag), and strontium (Sr) [1].

Although azide and lactate are not normally found in water, these compounds were added to the standards for additional possible detection. Azides are used in chemical industry as propellants and detonators and are precursors to amines [2]. Lactate is the conjugate base of lactic acid, which is used in topical preparations, cosmetics and detergents, found in milk products, and used as a food additive, among other uses [3]. Lactate could possibly be present in wastewater influent due to the usage and disposal by the general population as well as industry.

Separation of an aqueous mixture of analytes by capillary electrophoresis (CE) is achieved *via* the electrophoretic mobilities of each analyte, which depend on the charge-to-size ratio. Typically, for successful anions separation, the normal cathode to anode direction of the electroosmotic flow (EOF) must be reversed [4]. A variety of cationic surfactants such as tetradecyltrimethylammonium bromide, tetraethylenepentaamine,1,6-bis-(trimethylammonium)hexane, and 1,3,5-benzenetricarboxylic acid have also been used to enhance separation [5,6]. The cationic surfactants bind to the negatively charged wall of the capillary effectively creating a positively charged surfactant bi-layer and reversing the EOF direction. A negative voltage is then applied for analysis as both the EOF and the anions migrate in the same direction [4].

Because many anions do not have chromophores, detection must be achieved using indirect UV detection methods whereby compounds that absorb in the UV region and have high mobility are added to a buffer solution referred to as the background electrolyte (BGE). Several examples of indirect UV detection being implemented in the detection of anions exist in literature [7–13]. In brief, a UV-absorbing solute of the same charge is added to the BGE and serves as the visualizing reagent. When solute ions migrate past the detector window, they are measured as negative peaks relative to the absorbing solute. By reversing the signal and reference wavelengths of the diode-array detector, a positive signal is obtained whereby the area of the peak generated is linearly related to the sample concentration [4]. The migration speed of the UV-absorbing ion must closely match that of the ion being detected otherwise the analyte peak shape is severely distorted resulting in poor sensitivity. Depending on the size of the anion, different low concentration additives generating low currents (i.e. 5–10 μA) have been used for optimal peak shape and sensitivity [4,5,14]. Alternatively, zwitterionic buffers such as TRIS are often used to prevent excessively noisy baselines due to increased joule heating in the capillary [4].

The U.S. Environmental Protection Agency (EPA) has set regulations and parameters for various ions and compounds found in potable water [15]. Constituents existing in all natural waters include bicarbonate/carbonate, chloride, silicate and sulfate, but of these only chloride and sulfate are regulated by the EPA. Other regulated constituents found in water include arsenic/arsenate, bromide/bromate, fluoride, nitrite, nitrate, and phosphate, which arise from erosion and dissolution of minerals, ores and natural deposits, oxidative or other chemical reactions, agricultural and industrial run-off, sewage, or purposeful additions. Presence of regulated constituents in excess of the maximum contaminant levels (MCLs) can cause health issues including damage to major organs, cancer and/or mutations in DNA, diarrhea and dehydration, and various other effects depending on the constituent. Table 1 summarizes the tested anions/constituents that could be found in potable and natural water systems, EPAs regulation standard (if applicable), the possible sources or causes of the constituent, and the significance towards health of the constituent.

**Figure 1:**
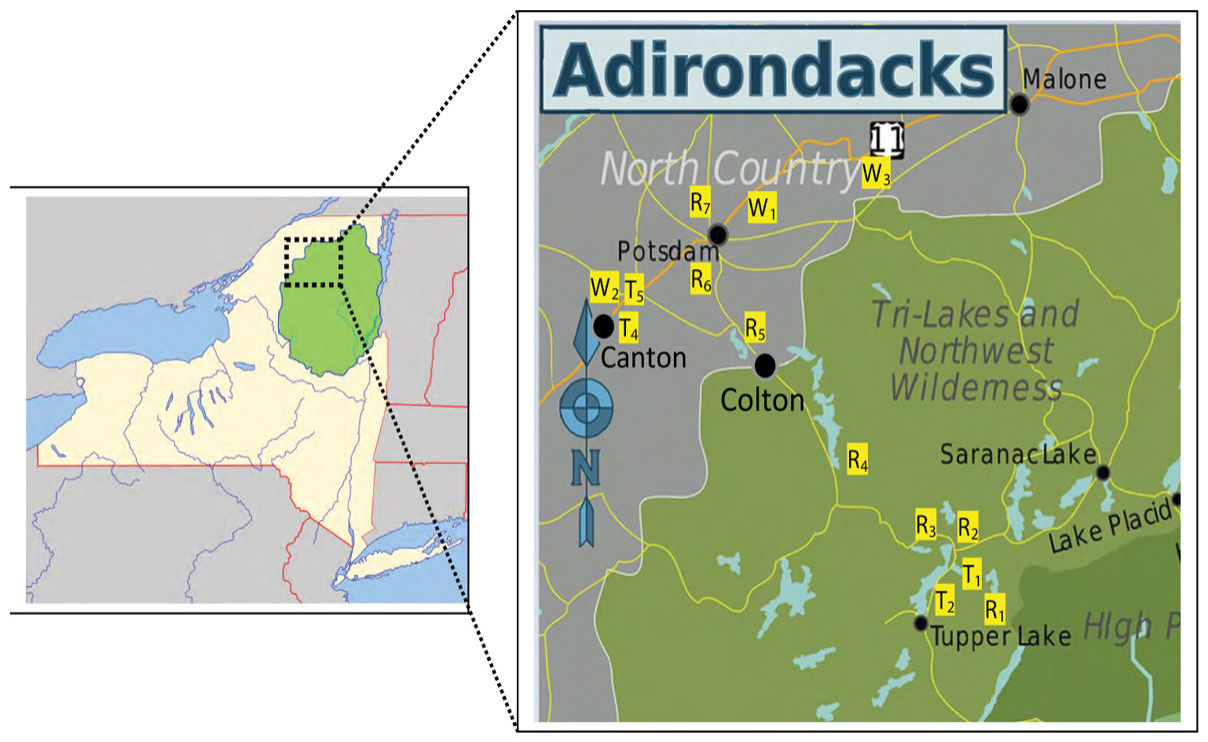
**Left map:** Map of New York State and surrounding states in the US, southeastern Canada with dashed-lined box representing the collection site area. **Right map:** Sample site map of Raquette River water system sites, including pre- and post-treated waste water from Tupper Lake and Potsdam, (R), tap water sites (T), and well water sites (W) in northern New York State, USA. The left image is credited to Jackaranga and Daniel Case under the license “Creative Commons CC BY-SA 4.0”. The right image is credited to Peter Fitzgerald, Jackaranga, Algorerhythms, and Daniel Case under the license “Creative Commons CC BY-SA 4.0”.

Capillary electrophoresis has been employed in recent years to examine ions in water and other media [7–13,16–20]. Particular interest is in capillary zone electrophoresis (CZE), an attractive separation method based on mass to charge ratios and the differences in mobilities among the various ions. In the present work, CZE with indirect UV detection is used to determine the presence and concentration of various anions in water samples taken in the Northern New York area, primarily of the Raquette River water system, which is the third longest river in New York State and the largest watershed of the central and Western Adirondacks. The river system begins at Blue Mountain Lake, flows south to Raquette Lake, turns Northeast to Long Lake, heads North to Tupper Lake through the Adirondack Park before it finally empties into the St. Lawrence River, Northeast of Massena and borderline with Canada (Figure 1). The Raquette River is one of the most popular, recreationally traveled and fished rivers of the Adirondack Park. Historically, every community along the River drew their drinking water from this river system; but due to changes the water quality of this watershed, only a handful of communities still draw their drinking water out of this source. The rest have been forced by the New York State Department of Health (NYSDOH) regulations on water quality to obtain their drinking water from deep-water wells. The village of Potsdam in Upstate New York resides on the banks of the Raquette River and is the largest user of drinking water drawn off the River in addition to two local universities, State University of New York at Potsdam and Clarkson University.

## Materials and Methods

### Chemicals

Sodium azide, sodium bromide, sodium chloride, potassium fluoride, potassium nitrate, sodium phosphate dibasic, sodium silicate, sodium sulfate, and sodium thiosulfate were obtained from Fisher Scientific (Fairlawn, NJ, USA). Sodium acetate, sodium arsenate, sodium carbonate, and potassium nitrite were purchased from J.T. Baker (Phillipsburg, NJ, USA). Lactic acid was acquired from Eastman (Kingsport, TN, USA). All reagents were of analytical grade. Ultra-pure CE water procured from Agilent Technologies (Santa Clara, CA, USA) from their Forensic Anion Solution Kit (PN 5064-8208).

### Standards and BGE

Agilent’s Inorganic Anion Test Mixture stock (1000 ppm fluoride, chloride, bromide, nitrite, nitrate, sulfate, and 2000 ppm phosphate) was diluted 1:10 with deionized (DI) water obtained from EMD Millipore Elix10 Water Purification System (Darmstadt, Germany) and used as a standard. Stocks of the following inorganic anions (from their sodium or potassium salts) and organic acids (i.e., lactic acid) were made at 1000 ppm: acetate, arsenate, azide, bicarbonate/carbonate, bromide, chloride, fluoride, lactate, nitrate, nitrite, phosphate, silicate, sulfate, and thiosulfate. Each anion and organic acid was diluted from its stock solution to make a 20 ppm standard mixture and was filtered using a 0.22 μm PES syringe filter (Argos Technologies, Elgin, IL, USA). The running buffer/BGE was Agilent’s proprietary Basic Anion Buffer (pH 12.0) obtained from their Forensic Anion Solution Kit. The BGE was degassed for 10 min in a TA Instruments degassing station (New Castle, DE, USA) using 2 mL glass CE vials from Agilent Technologies prior to runs.

### Instrumentation

The capillary electrophoresis system used in this study is the Agilent G7100 with a UV-DAD (diode array detector) interfaced with ChemStation software for data acquisition (Agilent Technologies, Santa Clara, CA, USA). The separation capillary used was a standard bare fused silica from Agilent Technologies, measuring 112.5 cm total length, 104 cm effective length, and an internal diameter (id) of 50 μm. Baseline noise is markedly increased if a wider internal diameter capillary is used due to the high UV absorptivity of the buffer.

### Procedures

Prior to first use, the capillary was flushed with running buffer/BGE for 30 min. Daily conditioning of capillary included 20 min flush of BGE. The capillary was conditioned between runs for 2 min using BGE from outlet Home vial and another 2 min of BGE from inlet Home vial. No sodium hydroxide was used in the conditioning of the capillary due to determined degradation of separation quality, reproducibility and performance with this application method [20]. Conditioning of the capillary at the end of the day involved 15 min ultra-pure water flush followed by 3 min air flush. The applied voltage was 30 kV negative polarity and the capillary cassette was kept at 30°C for all experiments. Negative polarity is used since the EOF is reversed. Sample injection was performed under Hydrodynamic injections of 50 mbar for 6 s followed by buffer injections of 50 mbar for 4 s. UV-vis signal detection was performed at 350 nm (20 nm bandwidth) with an indirect reference signal of 275 nm (10 nm bandwidth) and a runtime of 15 min. This procedure was based on Agilent’s Forensic Anion Solution Kit procedure manual [21].

### Samples

All water samples (natural river waters, potable tap and well waters, and pre- and post-treatment wastewaters) were collected in BPA-free plastic gallon water containers and filtered through a 0.22 μm PES syringe filter. No further manipulation of water samples (i.e., dilution, concentration, pH adjustment, complexation, etc.) was performed so as to mimic natural water conditions during testing. A set of samples were stored at 4°C and another set of same samples were kept at approximately 20°C. Samples were tested within 2 weeks of collection. Water samples were obtained from water sources feeding areas of interest, mainly Raquette River system from Tupper Lake (TL), NY, to Potsdam, NY, as well as pre- and post- wastewater treatment (WWT) from the two villages. Tap and well waters from other local areas including Canton and Winthrop, NY, were also tested. Figure 1 depicts Northern New York map of sample site location.

## Results and Discussion

### Anion analysis of standard mixtures

**Figure 2:**
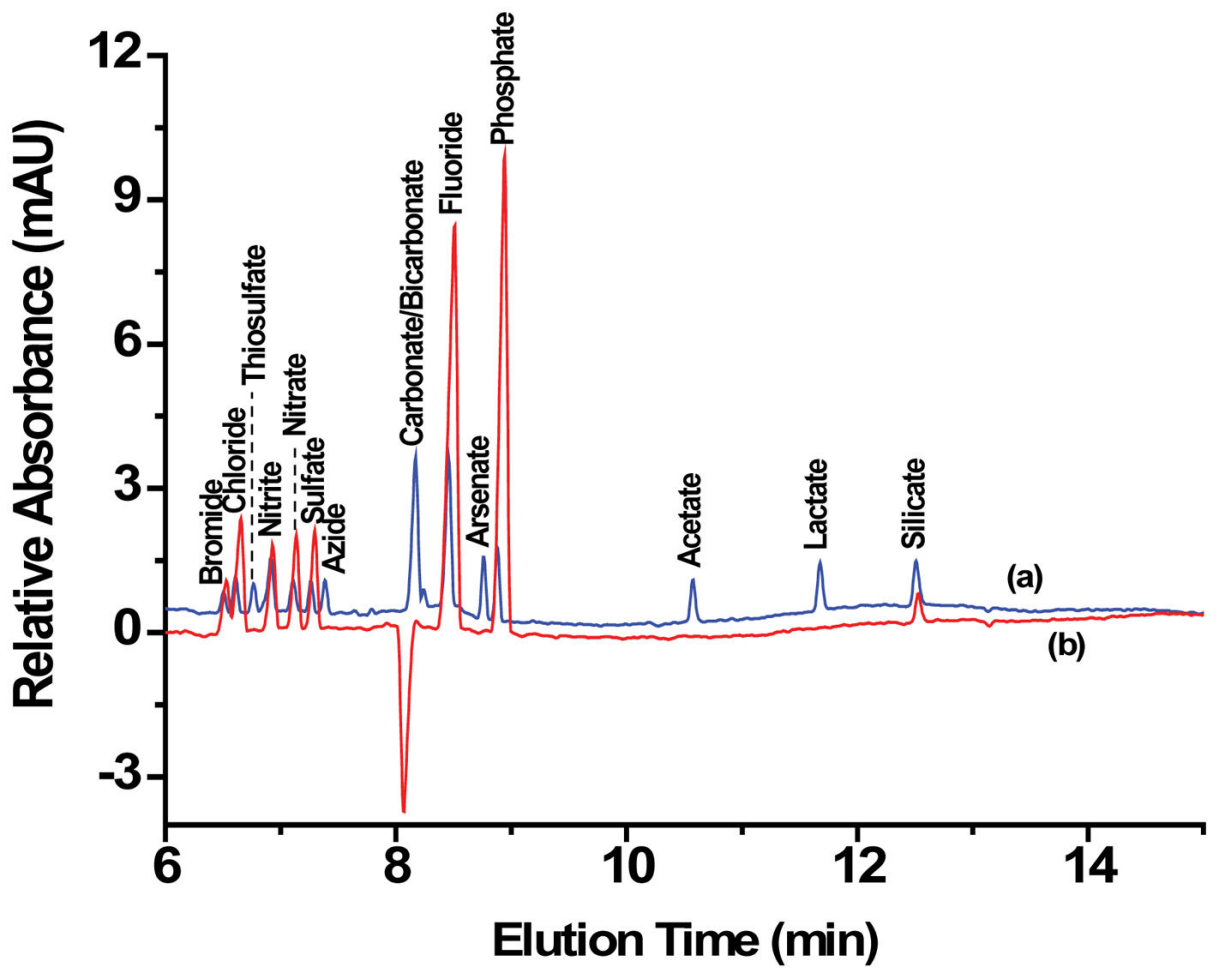
Electropherograms of two separate reference anion standard mixtures. **(a)** The blue electropherogram is that of fourteen-anion standard mixture prepared by us in the lab. The concentration of all anions (bromide, chloride, thiosulfate, nitrite, nitrate, sulfate, azide, fluoride, arsenate, phosphate, acetate, lactate, and silicate) was 20 ppm each whereas that of bicarbonate/carbonate was at 40 ppm. **(b)** The red electropherogram is that of seven-anion standard mixture from Agilent Technologies. The concentration of the anions bromide, chloride, nitrite, nitrate, sulfate, and fluoride was at 100 ppm each and that of phosphate was at 200 ppm. The background electrolyte (BGE) was Agilent’s proprietary Basic Anion Buffer (pH 12.0). Separation conditions: 30 kV negative polarity, 30°C, hydrodynamic injection of 50 mbar for 6 s with post injection of buffer at 50 mbar for 4 s, UV-vis signal detection at 350 nm with indirect reference of 275 nm.

Figure 2 shows the electropherograms of (a) fourteen-anion standard mixture using the sodium or potassium salts of each inorganic anion, and lactic acid for the organic anion, lactate and (b) seven inorganic anion standard mixture from Agilent Technologies. The standard mix from Agilent consisted of inorganic anions commonly monitored in water (bromide, chloride, nitrite, nitrate, sulfate, fluoride, and phosphate). The additional seven anions in our standard mix include azide, arsenate, acetate, lactate, silicate and thiosulfate. As can be seen in Figure 2, the electropherograms of eluted anions from the two different sources are identical suggesting high reproducibility and robustness of the separation method. On the basis of CZE separation, bromide was the first ion out of the seven commonly monitored inorganic anions to elute followed by chloride, nitrite, nitrate, sulfate, fluoride, and then phosphate. Resolution between bromide and chloride can be improved by decreasing operating temperature to 20 °C, but was not necessary for detecting and quantifying the two anions in our tested samples. All seven ions eluted under 9 min. The detection of silicate found in the Agilent Anion Standard mix electropherogram (Figure 2b) was the result of silicate being present in our DI water which was used to dilute the concentrated Agilent standard mix. The anions from our fourteen-anion standard mix migrated in the following order: bromide, chloride, thiosulfate, nitrite, nitrate, sulfate, azide, bicarbonate/carbonate, fluoride, arsenate, phosphate, acetate, lactate, and silicate, all eluting under 13 min.

**Figure 3:**
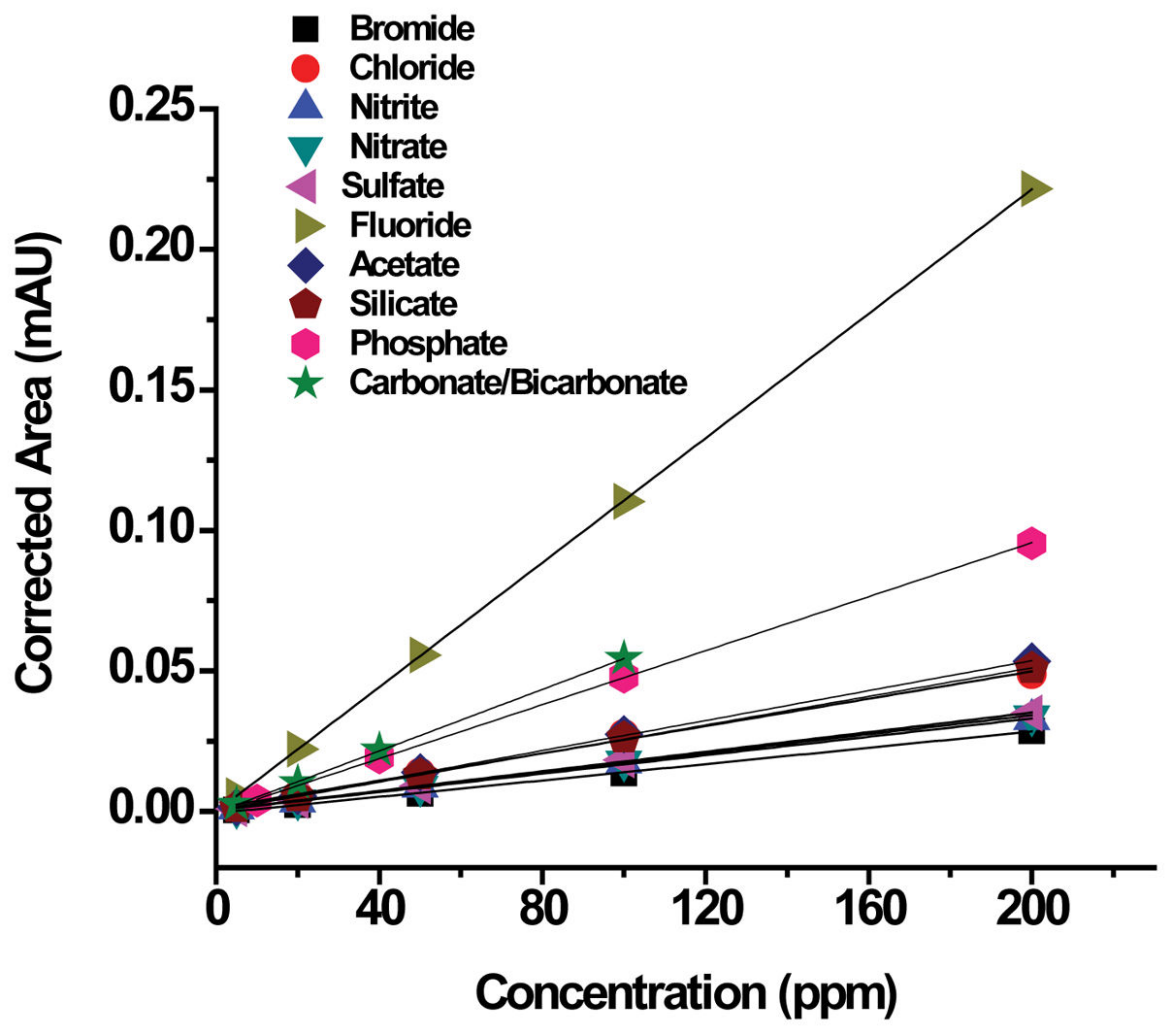
Calibration curves for ten anion standards with concentrations of 5, 20, 50, 100, and 200 ppm for bromide, chloride, nitrite, nitrate, sulfate, fluoride, acetate, and silicate; 10, 40, 100, and 200 ppm for phosphate; and 5, 20, 40, and 100 ppm for carbonate/bicarbonate.

To quantify the amount of detected anions in the water samples, a series of anion standards calibration curves were created (Figure 3). The concentration of the standard anion solutions of bromide, chloride, nitrite, nitrate, sulfate, fluoride, acetate, silicate, phosphate, and bicarbonate/carbonate varied between 5 and 200 ppm. Only calibration curves for the ten anions detected in the water samples are being presented in Figure 3. The goodness of the fit is determined from the coefficients of determination, R-squared (R^2^), which ranged between 0.99817 and 1.00000 as displayed in Table 2.

Table 2 displays the values for the propagation of the detected anion standards calibration curves (Figure 3). The curves were generated via amount in parts per million (ppm) by corrected area in milli-Absorbance Units (mAU). The corrected area was calculated by the area, which is absorbance x time (mAU × min), divided by migration time in min. All values for the calibration table, linear regression equations and subsequent calibration curves were auto-generated using the ChemStation program. The limits of detection (LOD) for all analytes were in the range of 3–5 ppm with 300 mbar pressure injection with a signal to noise ratio of 3.

### Anions analysis from natural and wastewater sources

**Figure 4:**
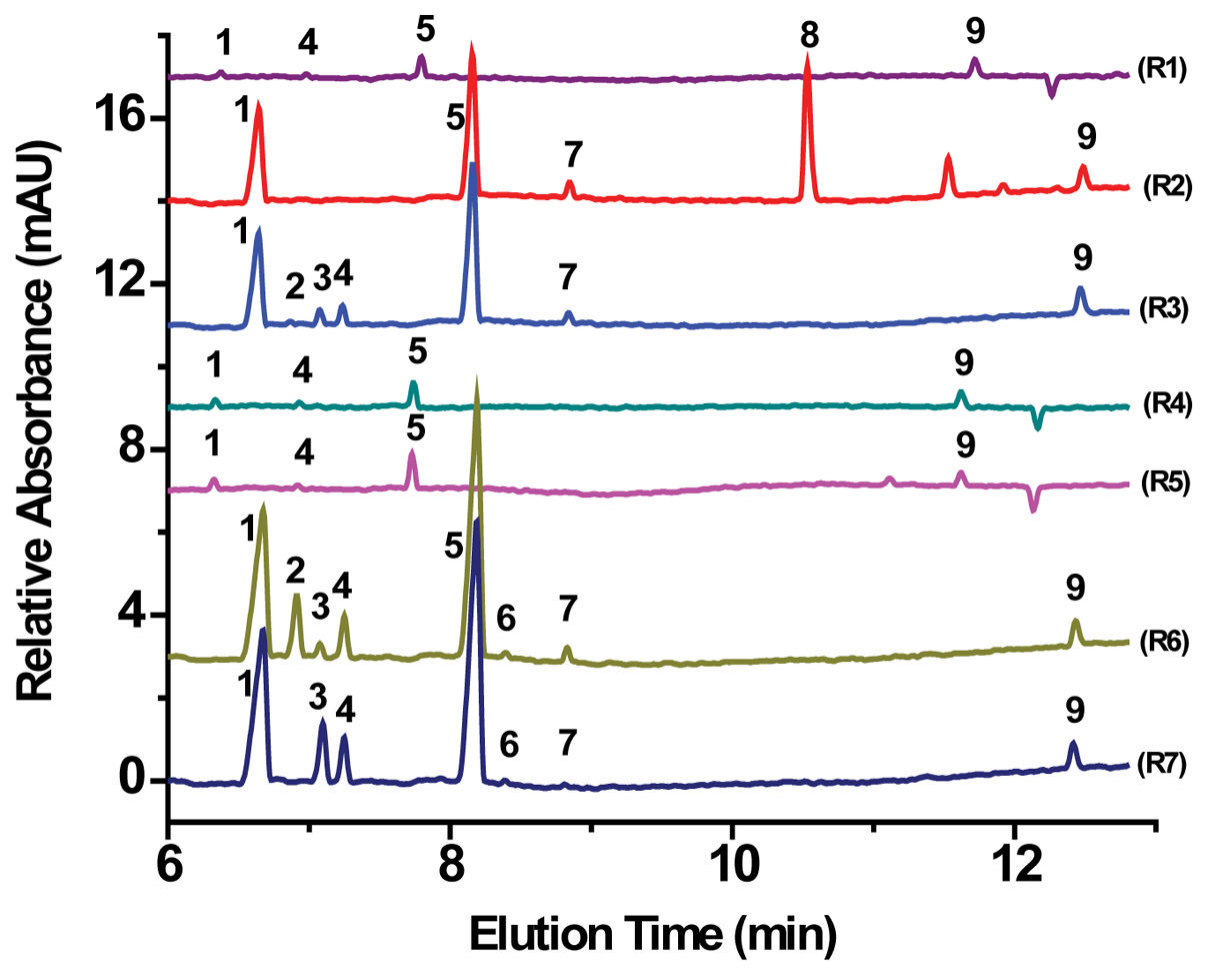
Electropherograms of water samples from (R1) Raquette River at the Crusher, (R2) Pre-WWT in Tupper Lake, (R3) Post-WWT in Tupper Lake, (R4) Carry Falls Reservoir, (R5) South Colton Reservoir, (R6) Pre-WWT in Potsdam, and (R7) Post-WWT in Potsdam. Conditions as in Figure 2 Peaks: 1-Chloride; 2-Nitrite; 3-Nitrate; 4-Sulfate; 5-Bicarbonate/carbonate; 6-Fluoride; 7-Phosphate; 8-Acetate; 9-Silicate.

Natural water samples were obtained along the Raquette River watershed route, starting with Bog River Falls, the site closest upstream to the source (Blue Mountain Lake), following downstream as far north, heading toward the St. Lawrence River, as Potsdam, NY. The seven sites for which the electropherograms in Figure 4 are displayed (R1–R7) represent the watershed and wastewater sites analyzed in this study. All tested samples contained chloride, bicarbonate/carbonate, and silicate, and most samples contained sulfate. Arsenate, azide, bromide, lactate, and thiosulfate were not detected in any of the samples. The only anions detected in the natural water samples from Raquette River sites depicted in Figure 4 and located at the Crusher (R1), Carry Falls Reservoir (R4), and South Colton Reservoir (R5) were chloride, sulfate, bicarbonate/carbonate, and silicate. Acetate was detected in the wastewater in Tupper Lake (Figure 4, R2) but not in the wastewater in Potsdam (Figure 4, R6) although it is used in the denitrification process in wastewater treatment solutions. Interestingly, nitrite and nitrate were both present in the wastewater effluent in Tupper Lake (R3), but not in Tupper Lake’s wastewater influent (R2), whereas Potsdam’s wastewater influent (R6) does contain nitrite and nitrate, but Potsdam’s wastewater effluent (R7) shows no nitrite and an increase of nitrate. The peaks between acetate (8) and silicate (9) in sample R2 and phosphate (7) and silicate (9) in sample R5 in Figure 4 remained unidentified.

### Potable water analysis

**Figure 5:**
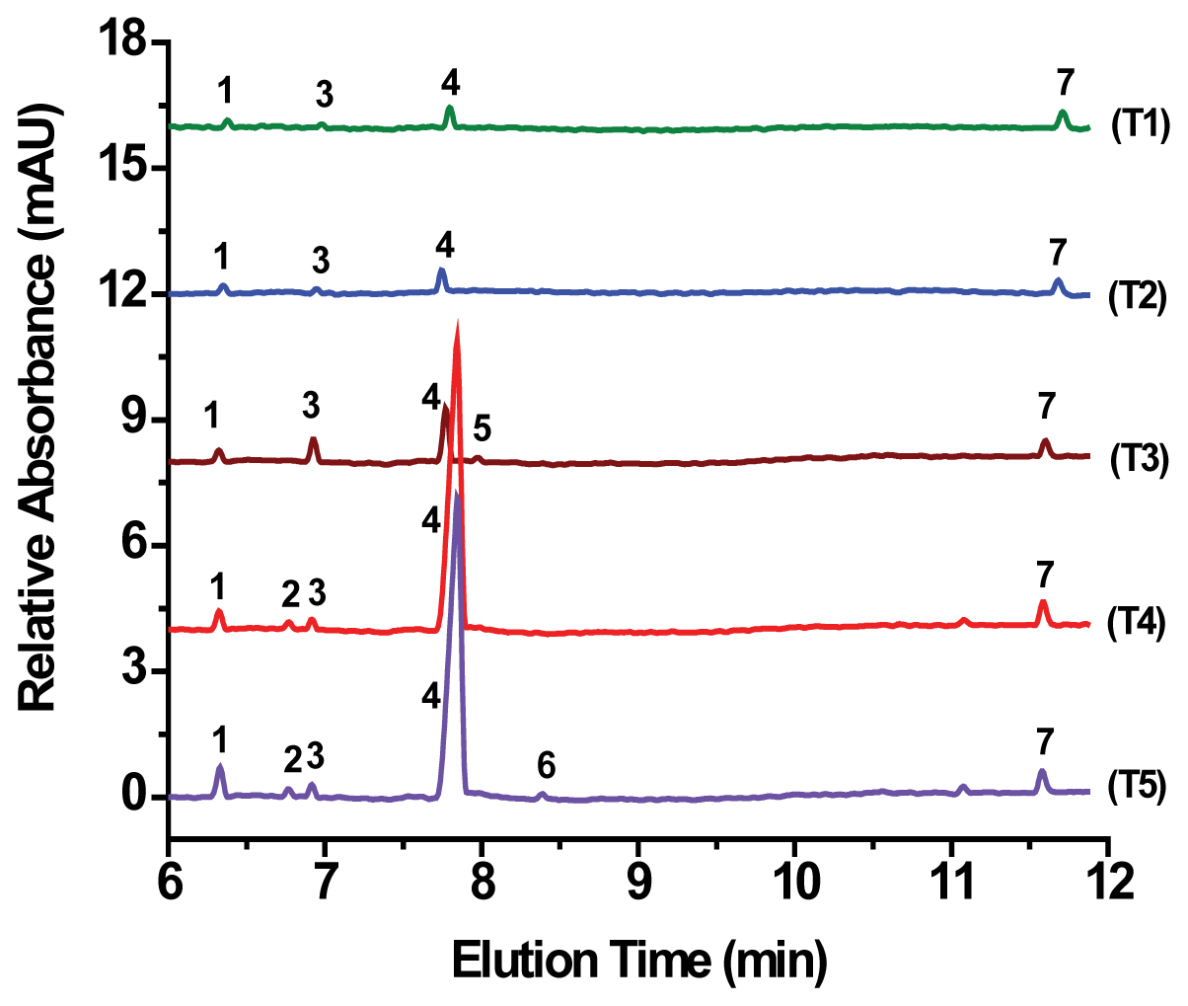
Electropherograms of tap water samples from (T1) Co-op located in village of Tupper Lake, (T2) a home located on Mt. Morris in Tupper Lake, (T3) State University of New York at Potsdam, (T4) St. Lawrence University, and (T5) home in village of Canton. Conditions as in Figure 2 Peaks: 1-Chloride; 2-Nitrite; 3-Nitrate; 4-Bicarbonate/carbonate; 5-Fluoride; 6-Phosphate; 7-Silicate.

**Figure 6:**
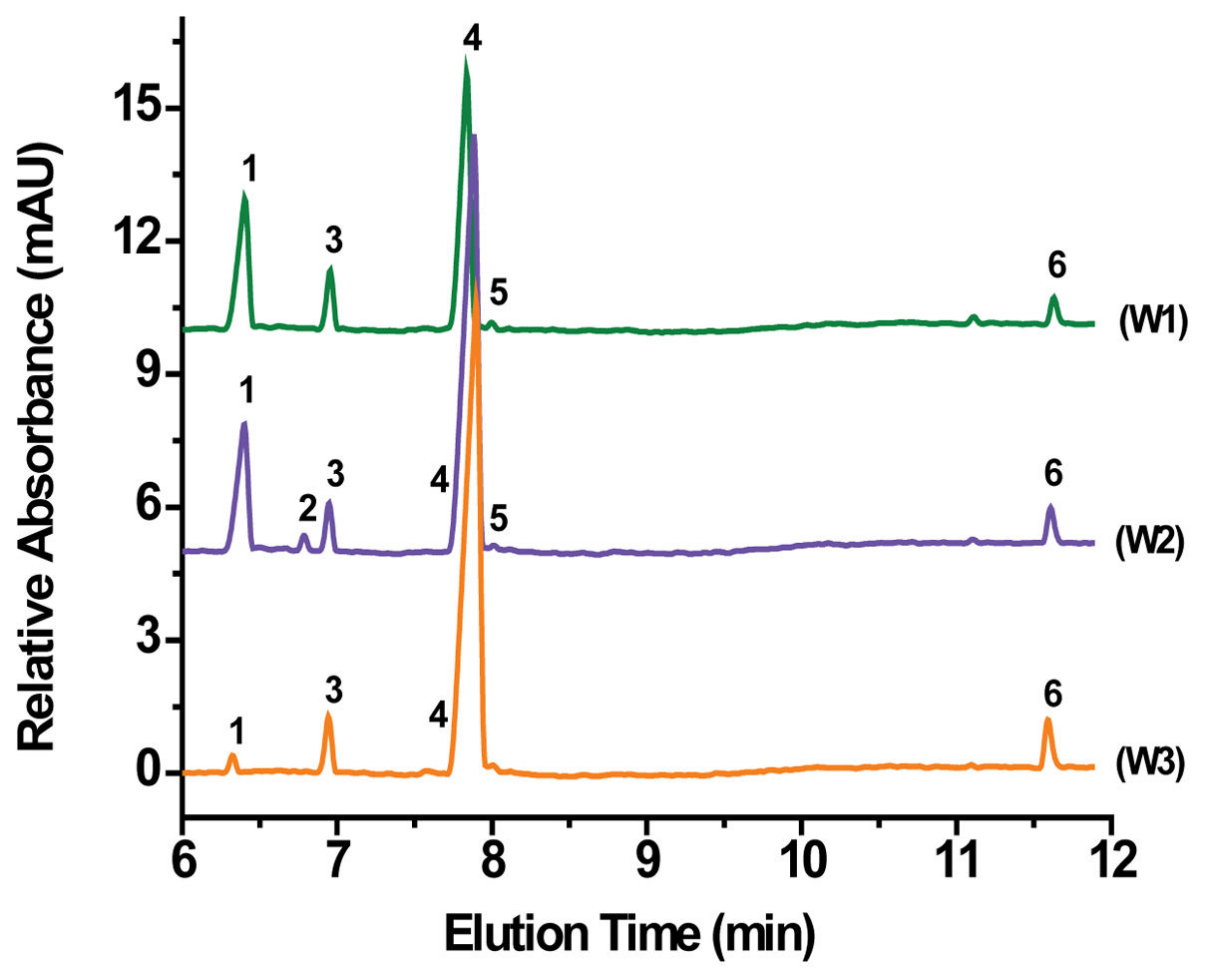
Electropherograms of well water samples from (W1) home in town of Potsdam, (W2) home in town of Canton, and (W3) home in village of Brasher Falls-Winthrop. Conditions as in Figure 2 Peaks: 1-Chloride; 2- Nitrate; 3-Sulfate; 4-Bicarbonate/carbonate; 5-Fluoride; 6-Silicate.

Potable water samples from taps and wells were obtained from various homes and institutions in and around Canton, Potsdam, Tupper Lake, and Brasher Falls-Winthrop, NY. All tap and well water samples contained chloride, sulfate, nitrate, bicarbonate/carbonate, and silicate (Figures 5 and 6). Table 3 displays the sites, locations, and concentrations in ppm of all the anions detected in the water samples. An expected small amount of fluoride, <5.0 ppm (estimated value to be <1 ppm based on linear extrapolation), was detected in the tap water obtained from SUNY Potsdam (Figure 4 and Table 3, T3) since the water treatment facility for the Village of Potsdam fluoridates the water supply. Unexpectedly, fluoride was also detected at <5.0 ppm (estimated value to be <1 ppm based on linear extrapolation), in the well water samples of the Town of Potsdam (Figure 6, W1) and the Town of Canton (Figure 6, W2). However, the estimated concentrations of fluoride (based on linear extrapolation) found in the analyzed samples fall under the EPA Maximum Contaminant Level of 4.0 ppm (Table 1). All tap water samples represented in Figure 5 (T1–T5 samples ) contained chloride, sulfate, silicate, and bicarbonate/carbonate with the two tap from Canton (T4 and T5) having the highest amount of bicarbonate/carbonate at 135 and 141 ppm, respectively. Furthermore, the two tap samples from Canton (T4 and T5) contained small amounts of nitrate (<5.0 ppm, Table 1) with sample T5 showing a small amount of phosphate (<5.0 ppm).

The well water samples obtained from homes in the Town of Canton (Figure 6, W2) and village of Brasher-Falls/Winthrop (W3) areas had the highest bicarbonate/carbonate concentrations of all samples tested, 217 and 260 ppm, respectively. This is not remarkably unusual since the geologic region is known to produce hard water for those who have wells as their potable water source. Sulfate and carbonate, in combination with calcium and magnesium cause water hardness and is typically found in natural waters. Even these high concentrations of anions fall within normal levels as dictated by the EPA (Table 1). Nitrate concentration at 11 ppm was detected in one well water sample in the Town of Canton, a level just over the EPA’s MCL value. This rather high nitrate concentration could be due to either agricultural fertilizer run-off, given that many Northern New York communities (including Canton, NY) are dedicated to farming and agriculture, leaking from a septic tank/sewer, natural deposits erosion, or any combination of the three. No bromide, thiosulfate, azide, arsenate, and lactate were detected in any of the water samples tested. However, a more systematic study involving hundreds of samples from older homes is needed to rule out the presence of any of these anions in the water supply.

Samples tested from the same sources stored at different temperatures, 4°C and 20°C, were not found to have significant differences in findings regarding detection or quantification.

## Conclusions

Our study showed that capillary zone electrophoresis (CZE) with indirect UV detection is a sensitive, reliable, and suitable method for the determination of many inorganic and organic anions. The simple, fast and cheap sample preparation method makes CZE an attractive tool for the detection and quantification of several anions present in wastewaters, natural waters, and potable waters including tap and well waters. Under our experimental conditions, all tested anions eluted within 12 min with excellent linearity and reproducibility. There were no significant differences in results from the water samples stored in 4°C versus same samples stored at 20°C. The concentrations of tested EPA regulated anions were found to be within acceptable limits although the presence of fluoride in some of the water samples tested is a concern and high levels of bicarbonate/carbonate or sulfate in combination with calcium and magnesium contribute to water hardness.

## Figures and Tables

**Table 1:** Water-quality criteria, standards, or limits for selected properties and constituents. All regulated standards are from U.S. Environmental Protection Agency, EPA [15].

Constituent	Standard	Source or Cause	Significance
Acetate	---	Used as carbon source in denitrification process.	Health effects vary depending on what it is compounded with.
Arsenic/Arsenate	10 μg/L MCL	Dissolution of minerals and ores, from industrial effluents, and from atmospheric deposition.	Toxic to humans and animals. A cumulative poison that is slowly excreted. Can cause nasal ulcers; damage to the kidneys, liver, and intestinal walls; and death; carcinogenic.
Bicarbonate/Carbonate	---	Carbon dioxide dissolved by naturally circulating waters; represents linkage between carbon cycle and hydrologic cycle.	In combination with calcium and magnesium forms carbonate hardness.
Bromide/Bromate	10 μg/L MCL	Bromate occurs when bromide ions present in water are oxidized by ozone and some other oxidizing agents (including, it is believed, chlorine)	Bromate is both carcinogenic and mutagenic.
Chloride	250 mg/L SMCL	Exists in all natural waters; In soil and rock formations, sea spray, waste discharges, and industrial brine. Sewage contains large amounts of chloride, as do some industrial effluents.	Does not pose a health hazard to humans; principal consideration is related to palatability. Large concentrations increase the corrosiveness of water and, in combination with sodium, give water a salty taste.
Fluoride	4.0 mg/L MCL 2.0 mg/L SMCL	Occurs naturally in rare instances; arises almost exclusively from fluoridation of public water supplies and from industrial discharges.	Potential health effects of long-term exposure to elevated fluoride concentrations include dental and skeletal fluorosis, nephrotoxicity, and may affect neurodevelopment in children.
Nitrite (mg/L, as N)	1 mg/L MCL	Commonly formed as an intermediate product in bacterially mediated nitrification and denitrification of ammonia and other organic nitrogen compounds. Typically from untreated or partially treated wastes.	An acute health concern at certain levels of exposure. Concentrations greater than 1.0 mg/L, as nitrogen, may be injurious to pregnant women, children, and the elderly. Excess exposure can cause methemoglobinemia, or blue-baby syndrome, anemia and preeclampsia in pregnant women.
Nitrate (mg/L, as N)	10 mg/L MCL	Oxidation of ammonia: agricultural fertilizer run-off; leaking from septic tanks, sewage; erosion of natural deposits.	Concentrations greater than local background levels may indicate pollution by feedlot run-off, sewage, or fertilizers. Concentrations greater than 10 mg/L, as nitrogen, may be injurious to pregnant women, children, and the elderly. See nitrite for health effects.
Phosphate (mg/L, as P)	16 mg/L	Added to finished water to inhibit corrosion in distribution piping and residential plumbing. Phosphorus occurs widely in nature in plants, micro-organisms, and animal wastes. Widely used as agricultural fertilizer, major constituent of detergents, Run-off and sewage discharges.	No known adverse health effects.
Silicate	---	Always present in natural waters. Rocks and geologic formations.	No definite health implications in water.
Sulfate	250 mg/L SMCL	Rocks, geological formations, discharges and so on. Exists in all natural waters, concentrations vary according to terrain.	Sulfates of calcium and magnesium form hard scale. Large concentrations of sulfate have a laxative effect on some people and, in combination with other ions, give water a bitter taste.
Thiosulfate	---	Rapidly dechlorinates water, is notable for its use to halt bleaching in paper-making industry, used in smelting silver ore, producing leather goods, and in textile industry.	No known adverse health effects.

MCL: Maximum Contaminant Level; SMCL: Secondary Maximum Contaminant Level; mg/L: Milligrams per Liter (=ppm, parts per million); μg/L: Micrograms per Liter (=ppb, parts per billion); ---: No Limit Established.

**Table 2:** Raw data for the anion standards calibration curves with migration times.

Anion	Migration time (min)	Amount (ppm)	Corrected Area (mAU)	Correlation factor, R^2^	Linear regression equation
**Bromide**	6.376	5.00	4.2676E-4	0.99941	y=1.4558E-4x 4.578E-4
		20.00	2.3861E-3		
		50.00	6.3833E-3		
		100.00	1.3658E-2		
		200.00	2.8992E-2		
**Chloride**	6.449	5.00	1.5469E-3	0.99817	y=2.4561E-4x+8.679E-4
		20.00	5.7247E-3		
		50.00	1.3783E-2		
		100.00	2.7383E-2		
		200.00	4.8873E-2		
**Nitrite**	6.700	5.00	9.1049E-4	0.99963	y=1.6453E-4x+2.465E-4
		20.00	3.4472E-3		
		50.00	8.7465E-3		
		100.00	1.7255E-2		
		200.00	3.2821E-2		
**Nitrate**	6.894	5.00	1.0516E-3	0.99951	y=1.6954E-4x+3.851E-4
		20.00	4.0557E-3		
		50.00	8.9077E-3		
		100.00	1.7889E-2		
		200.00	3.3982E-2		
**Sulfate**	7.044	5.00	9.4979E-4	0.99967	y=1.7493E-4x+2.592E-4
		20.00	3.8510E-3		
		50.00	9.0739E-3		
		100.00	1.8359E-2		
		200.00	3.4920E-2		
**Fluoride**	8.189	5.00	5.4907E-3	1.00000	y=1.10725E-3x–2.64E-5
		20.00	2.2093E-2		
		50.00	5.5623E-2		
		100.00	1.1030E-1		
		200.00	2.2155E-1		
**Acetate**	9.965	5.00	1.4667E-3	0.99990	y=2.6727E-4x–2.565E-4
		20.00	5.5550E-3		
		50.00	1.3915E-2		
		100.00	2.7385E-2		
		200.00	5.3444E-2		
**Silicate**	11.649	5.00	1.3897E-3	0.99992	y=2.5482E-4x+1.766E-4
		20.00	5.2543E-3		
		50.00	1.2947E-2		
		100.00	2.6121E-2		
		200.00	5.0906E-2		
**Phosphate**	8.581	10.00	3.8703E-3	0.99999	y=4.7854E-4x–2.218E-4
		40.00	1.9228E-2		
		100.00	4.7902E-2		
		200.00	9.5435E-2		
**Carbonate/bicarbonate**	7.726	5.00	0.0254E-2	0.99878	y=5.6373E-4x–1.612E-3
		20.00	1.0159E-2		
		40.00	2.1784E-2		
		100.00	5.4371E-2		

**Table 3:** Concentration of anions (ppm) detected in the water samples tested in this study.

Site	Location	Concentration Levels (ppm)													
		Br^−^	Cl^−^	S_2_O_3_^2−^	NO_2_^−^	NO_3_^−^	SO_4_^2−^	N_3_^−^	CO_3_^2−^/HCO_3_^−^	F^−^	AsO_4_^3−^	PO_4_^3−^	C_3_H_3_O_2_^−^	Lactate	SiO_3_^2−^
**R1**	Raquette River, at Crusher	---	<5*	---	---	---	<5*	---	5.5	---	---	---	---	---	6.8
**R2**	Pre-WWT in Tupper Lake	---	95	---	---	---	---	---	47	---	---	<10*	56	---	8.4
**R3**	Post-WWT in Tupper Lake	---	98	---	<5*	10	14	---	59	---	---	<10*	---	---	10
**R4**	Carry Falls Reservoir	---	<5*	---	---	---	<5*	---	7.3	---	---	---	---	---	5.0
**R5**	South Colton Reservoir	---	<5*	---	---		<5*	---	9.4	---	---	---	---	---	<5*
**R6**	Pre-WWT in Potsdam	---	198	---	54	6.1	38	---	133	<5*	---	<10*	---	---	10
**R7**	Post-WWT in Potsdam	---	223	---	---	5.8	36	---	136	<5*	---	<10*	---	---	11
**T1**	Tap, village of Tupper Lake	---	<5*	---	---	---	<5*	---	5.4	---	---	---	---	---	6.3
**T2**	Tap, Mt. Morris in Tupper Lake	---	<5*	---	---	---	<5*	---	6.1	---	---	---	---	---	5.3
**T3**	Tap, village of Potsdam	---	<5*	---	---	---	18	---	14	<5*	---	---	---	---	6.1
**T4**	Tap, St. Lawrence Univ. in Canton	---	9.2	---	---	<5*	6.9	---	135	---	---	---	---	---	9.2
**T5**	Tap, village of Canton	---	19	---	---	<5*	9.1	---	141	---	---	<10*	---	---	8.6
**W1**	Well, Town of Potsdam	---	147	---	---	---	56	---	103	<5*	---	---	---	---	10
**W2**	Well, Town of Canton	---	143	---	---	11	42	---	217	<5*	---	---	---	---	14
**W3**	Well, Winthrop	---	8.4	---	---	---	51	---	260	---	---	---	---	---	20

* Reported values are based on the lowest concentration run of standards used in the calibration curves.
